# Workspace and trajectory-based experimental validation of a 3-armed 6-DOF parallel robot for femoral fracture surgery

**DOI:** 10.3389/frobt.2026.1819716

**Published:** 2026-05-29

**Authors:** Alberto Gaytan, Adnan Khaleeli, Marzieh Sadat Saeedi-Hosseiny, Charalampos Papachristou, Iulian Ioan Iordachita, Mohammad H. Abedin-Nasab

**Affiliations:** 1 Surgical Robotics Laboratory, Department of Biomedical Engineering, Rowan University, Camden, NJ, United States; 2 Robossis Inc., Cherry Hill, NJ, United States; 3 Department of Mathematics, Rowan University, Glassboro, NJ, United States; 4 Laboratory for Computational Sensing and Robotics, Department of Mechanical Engineering, Johns Hopkins University, Baltimore, MD, United States

**Keywords:** 6-DOF, bone alignment, femur fracture reduction, parallel robots, surgical robotics, trajectory testing, workspace analysis

## Abstract

Femur fractures remain a significant occurrence in the general population, requiring immediate medical care (typically surgery) followed by extensive recovery time. Femur fracture reduction, defined as the process of realigning femur bone fragments, is currently performed manually, a task that is physically demanding and associated with high malalignment rates. Our surgical system Robossis, a 3-armed, 6-DOF parallel robot, is aimed at addressing these issues by exerting the necessary forces to eliminate the physical demand while facilitating proper fracture reduction. The new Robossis V2 system demonstrates improvements over our previous V1 system in both clinical usability and movement capabilities. In this work, we present an experimental validation of Robossis V2 through numerical workspace analysis and optical-tracking-based trajectory testing. Workspace evaluation shows that Robossis V2 provides substantial coverage beyond clinically required alignment ranges, with constrained translational and rotational workspace volumes approximately 136 times and 79 times larger than the required range, respectively. Across all collected trajectory data, Robossis V2 achieved consistent sub-millimeter translational and sub-degree rotational performance, with the 75th percentile of absolute errors remaining below 1 mm and 1 ° . These results establish the viability of Robossis V2 as a clinically promising platform for high-precision femoral fracture reduction.

## 1 Introduction

Long-bone fractures account for more than half of all fractures worldwide (Court-Brown and Caesar, 2006; Rennie et al., 2007). Globally, an estimated 2.9 million femur fractures occur annually due to motor vehicle accidents alone (Agarwal-Harding et al., 2015; Johnell and Kanis, 2006; Melton III et al., 1998; Kanis et al., 2000; Gullberg et al., 1997 Cooper et al., 1992). In the United States, approximately 430,000 femur fracture surgeries are performed each year (Ng et al., 2012; Enninghorst et al., 2013; Cooper et al., 1993; Leibson et al., 2002; Magaziner et al., 1997; Magaziner et al., 1990; Riggs and Melton Iii, 1995;Kannus et al., 1996). With an aging population, incidence is projected to increase at a compound annual growth rate of 5% and is expected to double by 2050 (Ng et al., 2012; Court-Brown and Caesar, 2006; Rennie et al., 2007).

Standard treatment involves three primary steps: (1) positioning the patient under a C-arm to acquire intraoperative fluoroscopic images (Saeedi-Hosseiny et al., 2025); (2) manually reducing the fracture through iterative trial-and-error maneuvers; and (3) stabilizing the bone using intramedullary nails or plates (Wolinsky et al., 1999; Gosling et al., 2005; Buschbaum et al., 2015; Garrison et al., 2021; Kumar et al., 2020; Shui et al., 2021; Babhulkar et al., 2024). Although widely practiced, this workflow presents significant mechanical and imaging challenges.

Manual fracture reduction is particularly demanding. The femur’s length and the strong surrounding musculature require substantial traction forces and torques—reported averages of 517 N and 74 Nm (Gosling et al., 2005; Buschbaum et al., 2015; Jaarsma, 2004; Jaarsma et al., 2005; Westphal et al., 2009). Sustaining these loads can lead to surgeon fatigue, variability in applied force, overshoot, soft-tissue injury (Füchtmeier et al., 2004; Oszwald et al., 2008; Hawi et al., 2012), and malalignment-related complications. Furthermore, alignment is assessed primarily through intermittent 2D fluoroscopic imaging while attempting to achieve accurate 3D anatomical restoration. This mismatch often necessitates repeated imaging and multiple adjustment attempts, increasing radiation exposure for both patients and operating room staff.

Consequently, malalignment remains common. Rotational malalignment of 10° or more relative to the contralateral femur occurs in approximately one-third of patients (Sullivan et al., 2021; Abbas et al., 2023), with overall malalignment rates reported at 27.5% (Honeycutt et al., 2019). Improper alignment is associated with hip and knee pain, impaired mobility, leg-length discrepancy, abnormal gait, and an increased risk of degenerative arthritis (Jaarsma, 2004). Approximately 20% of patients require additional invasive procedures, increasing blood loss, hospital length of stay, cost, and complication risk (Drew et al., 2016; Lapidus et al., 2013; Schilcher, 2015). Among patients over 50 years of age, 1-year mortality following femur fracture surgery ranges from 20% to 26%, depending on fracture location (Liounakos et al., 2021; Moloney et al., 2016; Streubel et al., 2011; Wolf et al., 2020; Mattisson et al., 2018; Horner et al., 2017), with recent reports indicating rates near 24.9% (Cnossen et al., 2023).

Extensive customer discovery conducted through a National Science Foundation I-Corps program further highlighted this unmet clinical need. Interviews with 260 stakeholders—including trauma surgeons, hospital executives, and patients—consistently emphasized the challenges of achieving reliable alignment (Abedin-Nasab and Saeedi-Hosseiny, 2020). Surgeons described malalignment as occurring ”more than I care,” and estimated that the manual alignment portion of surgery alone requires 30–60 min. Many expressed strong interest in technologies capable of improving alignment reproducibility while reducing operative time and physical strain.

Together, these clinical and stakeholder insights underscore a significant unmet need for a surgical approach that enhances alignment accuracy, reduces the physical burden on the surgical team, and minimizes reliance on repeated fluoroscopic imaging.

To address these challenges, we introduce Robossis, a robotic surgical assistant designed to facilitate high-precision femoral fracture reduction. The system is engineered to manage the substantial forces required to counteract surrounding musculature while enabling controlled, repeatable fragment manipulation.

**FIGURE 1:**
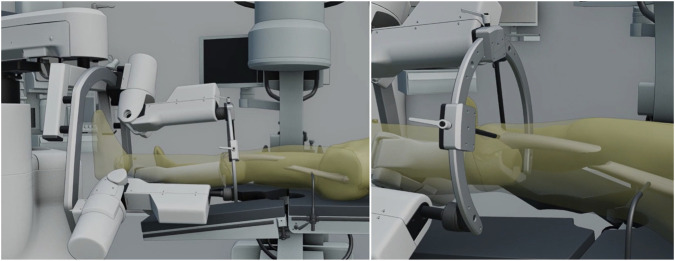
Simulated intraoperative configuration of the Robossis surgical system during femoral fracture alignment. A rendered representation of the robotic-assisted femoral fracture alignment setup is shown in a simulated operating room environment. The Moving Ring (MR) secures the distal femur segment, while the proximal fragment is stabilized via an external fixation assembly. The semi-transparent overlay illustrates the femoral anatomy and fracture configuration during manipulation. The parallel robotic manipulator enables controlled six-degree-of-freedom (6-DOF) repositioning of the distal fragment to achieve anatomical alignment under simulated surgical conditions.

In clinical terms, femoral fractures are typically conceptualized as two primary segments: a proximal fragment (closer to the torso) and a distal fragment (farther from the body). Robossis’s end-effector is attached to the distal fragment, which can be seen in Figure 1. The objective of robotic reduction is to transform the distal fragment into anatomical alignment with the proximal target. This is achieved through a multi-stage kinematic process. The first stage, extraction, resolves longitudinal overlap between fragments to establish a manipulable workspace. The robot then executes controlled translational and rotational adjustments to align the mechanical axes and advance the distal fragment toward precise cortical apposition.

## 2 Materials and methods

### 2.1 Design iteration and experimental validation

The development of Robossis V2 was guided by iterative experimental validation using both cadaver studies and simulation labs. The earlier V1 system was evaluated across three cadaver studies conducted in collaboration with five orthopedic trauma surgeons, as well as multiple simulation sessions involving a total of eight surgeons. These cadaver studies were reviewed and approved by Rowan University/Virtua Health IRB approval committee, under IRB Study G24066. The cadavers were acquired from the Robert Wood Johnson Foundation with the necessary consent for use in medical science.

These studies enabled direct observation of surgical workflows, identification of system limitations, and quantitative assessment of force requirements, workspace constraints, and usability challenges. Key limitations identified in V1 included restricted workspace and reduced flexibility in system positioning.

In the previous V1 configuration used with the cadavers, the three-armed parallel architecture distributed loads across the limbs, improving structural stiffness and enabling the V1 system to sustain traction forces of up to 1,000 N, which are clinically relevant for femoral fracture reduction (Rezapour-Shafigh et al., 2026). This greatly assisted surgeons in femur fracture reduction.

Insights from these experimental evaluations directly informed the mechanical and control design of the V2 system, resulting in improvements in workspace, adjustability, and stability.

### 2.2 Mechanical components

**FIGURE 2:**
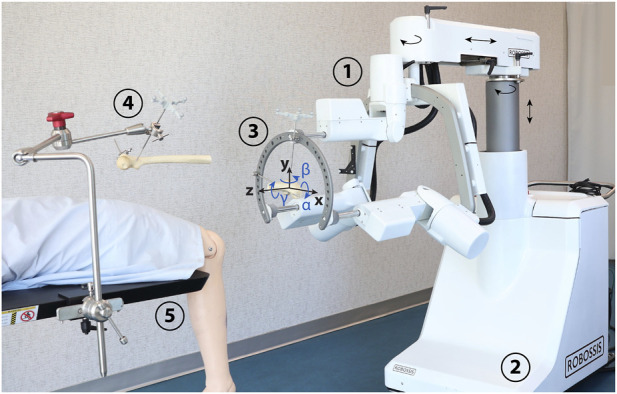
Robossis V2 setup and system degrees of freedom. The Robossis surgical system, integrated within the experimental femoral fracture validation setup, is shown. The robotic manipulator (1) is mounted on a mobile cart (2) and interfaces with the Moving Ring (MR) end-effector (3), which secures the distal femur specimen. The proximal fixation assembly (4) stabilizes the femoral shaft, while the mannequin limb and surgical Table 5 replicate operative positioning. The MR provides six degrees of freedom (6-DOF), enabling three translational (x, y, z) and three rotational ( α , β , γ ) motions for precise fracture reduction. The mobile cart provides four additional degrees of freedom (4-DOF), including vertical translation, horizontal translation, axial rotation, and base rotation, as indicated by arrows. This combined architecture allows coarse positioning via the cart and fine multi-axis manipulation via the parallel robotic manipulator during femur fracture alignment surgery.

The V2 system retains the core kinematic architecture of its predecessor while substantially expanding the operational workspace. Motion constraints are primarily governed by actuator stroke limits and mechanical boundaries specific to each configuration. Enhanced mobility features allow reorientation of the main robotic structure relative to the mobile cart, providing surgeons with increased flexibility in positioning. This dual-advantage approach yields both an expanded robotic workspace and improved accessibility for surgical teams. While Robossis has 6 DOF that can be actively controlled during operations, it has an additional 4 DOF that can be manipulated before the surgery to give the surgeon more flexibility. All these motions are depicted above the main cart body in Figure 2 (2).

#### 2.2.1 New vertical shaft

The vertical pillar to which the rings of the system are attached is capable of vertical movement in the range of 340 mm, a feature which was not present in the previous model. This allows for enhanced user experience as the initial setup is no longer constrained to a specific height. Movement of the vertical shaft can only be conducted during the initial patient configuration step of the system. This enhances safety, and any further vertical movement can be conducted through the manipulator. It is particularly useful when leveling Robossis with a surgical table or placing the rings over the patient.

#### 2.2.2 Robotic body rotation

The manipulator of the V2 system can be rotated at two separate points. The first is at the fixed ring, accessible via the handle directly above it. This can be loosened to allow rotation and then tightened at the desired rotation to lock it. The second is at the vertical shaft, also accessible via the handle directly above it. The same loosening and locking logic applies here. Again, these components introduce flexibility into the setup of the system, providing surgical teams with efficient usage.

#### 2.2.3 Robotic body extension

The system also allows horizontal translation of the manipulator along its connection to the vertical shaft. This is done using the handle located on the side of the horizontal connection piece (utilizing the same loosening and locking mechanism). Much like the other adjustable motions, this gives flexibility in setup where more or less distance between the robot and fractured leg is needed.

#### 2.2.4 Robot cart

All necessary electrical components for the robot itself are contained in the cart. The system can be moved in its entirety as needed, and locked into place using the floor locks. Weight is added inside the cart itself to prevent issues when attaching the system to a patient as well, an improvement over the previous version.

### 2.3 Impact of cadaver studies on mechanical design

Findings from cadaver studies and simulation labs played a central role in shaping the mechanical architecture of Robossis V2. Observations from V1 deployments highlighted the need for increased workspace adaptability and improved ergonomic positioning.

In response, V2 introduces several mechanical enhancements, including the addition of a vertically adjustable shaft, improved rotational and translational degrees of freedom, and a more stable and weighted cart system. These changes allow for more flexible system positioning relative to the patient and surgical table, addressing limitations encountered in earlier experimental use.

Furthermore, the system incorporates three robotic arms equipped with high-torque actuators, enabling the application of sufficient forces to accommodate a wide range of patient anatomies and fracture types. This design decision was directly motivated by force requirements observed during cadaver-based fracture reduction procedures.

### 2.4 Coordinate systems

Robossis V2, similar to its V1 predecessor, operates within the standard Cartesian coordinate system (x,y,z,α,β,γ) , where (x,y,z) represent translational degrees of freedom and (α,β,γ) denote rotational displacements about the X , Y , and Z axes, respectively.

To enhance clinical applicability, the system implements a surgical coordinate system optimized for orthopedic workspace navigation. This anatomical reference frame aligns with standard surgical orientations: medial/lateral, anterior/posterior, superior/inferior, extension/flexion, valgus/varus, and internal/external rotation. Since this study is focused on trajectory testing, we will be utilizing the standard engineering (Cartesian) units as it remains consistent across patient configurations. However, throughout this work, the patient is assumed to be in the supine position, under which the Cartesian reference frame is closely aligned to the surgical coordinate system.

**FIGURE 3:**
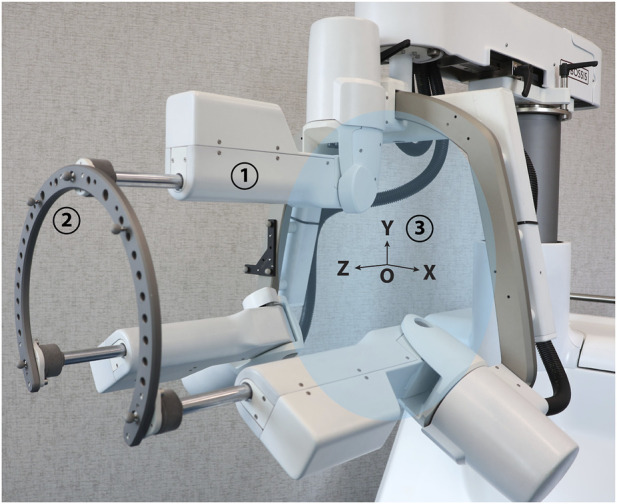
Robossis V2 3-armed parallel manipulator and defined Cartesian reference frame. The Robossis V2 reduction manipulator is shown with its three actuated arms (1) arranged in a parallel kinematic configuration converging at the Moving Ring (MR) (2). The shaded region (3) highlights the Fixed Ring (FR) area and the associated Cartesian coordinate system (X, Y, Z), with origin O defined at the geometric center of the FR.

All trajectory commands are defined relative to the coordinate origin shown in Figure 3 (3). The robot’s operational workspace is defined as the set of all Cartesian configurations (x,y,z,α,β,γ) that the end-effector can physically attain without violating mechanical constraints.

Figure 3 also identifies key structural reference points within the system. The Moving Ring (MR) center serves as Robossis’s kinematic end-effector. During testing, measured positions obtained from the optical tracking system are compared directly to the commanded target positions at MR’s geometric center.

The Fixed Ring (FR) defines the global reference frame of the robot, with its origin located at (0 mm, 0 mm, 0 mm, 0 ° , 0 ° , 0 ° ). At the home configuration, the spatial relationship between the MR and FR is characterized by a pure linear translation along the Z-axis. Unlike the MR, which moves throughout operation, the FR remains stationary. All MR positions are therefore expressed relative to the FR coordinate frame. It is important to note that, unlike Robossis V1, the Fixed Ring in V2 is a virtual reference construct rather than a physical component.

### 2.5 Kinematics

Kinematics determines the required joint angles to achieve a desired pose of Robossis’s end-effector. For example, we send a command (x,y,z,α,β,γ) and the kinematics will provide the exact angles the motors need to rotate to achieve that pose. In this section, we discuss the equations related to the kinematics computation of Robossis and the parameters that define it.

In Robossis V2, actuation is provided by six primary motors: three small motors that drive the linear actuators and three large motors that set the corresponding rotary joint angles. The kinematic parameters used in this study are summarized in Table 1. Kinematically, Robossis V2 can be interpreted as a side-oriented variant of a Gough-Stewart platform: instead of six legs, the end-effector is supported by three actuated arms in a parallel configuration, and the pose-to-joint mapping follows the same general formulation used for Stewart-type mechanisms. The small motors extend or retract each arm, changing the arm lengths and producing end-effector motion. In particular, the arm located at γ=90° (top arm) primarily influences motion along the robot’s X -axis; as it rotates, the passive joints in the other two arms accommodate the induced load and the MR translates laterally. By contrast, coordinated extension of the two lower arms contributes more strongly to vertical motion in the robot frame, with passive joint compliance allowing the MR to elevate without lateral displacement.

**TABLE 1:** Kinematic parameters of Robossis V2.

Parameter	Robossis V2 values
Moving ring radius (h)	175.0 mm
Fixed ring radius (g)	175.0 mm
Length of arm $\left(d_{i}\right)$	365.00–565.00 mm
Arm position $\left(\gamma_{i}\right)$	−30°,90°,210°

**FIGURE 4:**
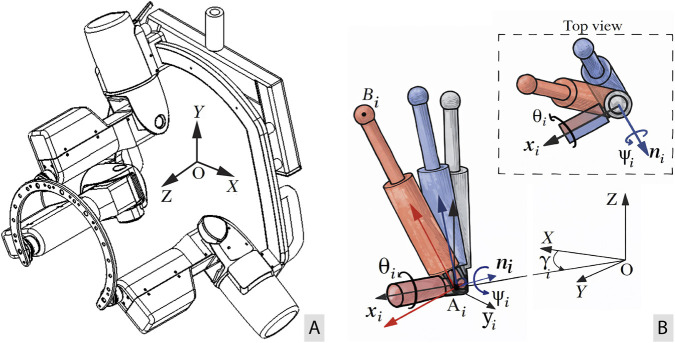
Robossis 3-armed parallel robot and kinematic parameters of its arms. **(A)** CAD model of the Robossis manipulator. **(B)** Kinematic representation of the i th arm and its associated coordinate frames. The universal joint variables of the i th arm are illustrated, where $\theta_{i}$ denotes the active rotation about the $x_{i}$ axis, followed by the passive rotation $\psi_{i}$ about the $n_{i}$ axis. Points $A_{i}$ and $B_{i}$ indicate the base and platform attachment points, respectively, and O represents the origin of the global Cartesian reference frame at the center of the Fixed Ring (FR).

The first step is to convert the commanded pose into a rotation matrix. Robossis employs an intrinsic Euler XYZ convention, meaning the rotation axes are body-fixed and update after each successive rotation. Specifically, rotations are applied about X , then Y , and finally Z . Which is also equivalent to Euler ZYX extrinsic. This matrix represents the transformation from MR to FR, with FR’s origin being Point O in Figure 4A.

$${\;\;}^{FR}\;R_{MR}=\left[\begin{array}{ccc} \cos\beta \;\cos\gamma & -\cos\beta \;\sin\gamma & \sin\beta \\ \sin\alpha \;\sin\beta \;\cos\gamma +\cos\alpha \;\sin\gamma & -\sin\alpha \;\sin\beta \;\sin\gamma +\cos\alpha \;\cos\gamma & -\sin\alpha \;\cos\beta \\ -\cos\alpha \;\sin\beta \;\cos\gamma +\sin\alpha \;\sin\gamma & \cos\alpha \;\sin\beta \;\sin\gamma +\sin\alpha \;\cos\gamma & \cos\alpha \;\cos\beta \end{array}\right]$$ (1)

Now we can define vectors called $a_{i}$ , which represent the distance from the Fixed Ring frame origin (FR) to the i -th base attachment point $A_{i}$ , expressed in the Fixed Ring coordinate frame. The base attachment point is the point where one of the arms is connected to the base (fixed ring), as depicted in Figure 4B. Since Robossis has three arms, there will be three of these vectors. Similarly, we define ${\text{vectors}}^{MR}b_{i}$ that represent the attachment points $B_{i}$ on the moving ring (MR), expressed in the MR coordinate frame.

These constant vectors are given by Equation 2.

aiFR=gcosγisinγi0, MRbi=hcosγisinγi0. (2)

As illustrated in Table 1, g is the fixed ring (base) radius and h is the moving ring radius. Note in this case $\gamma_{i}$ represents the arm position for the three arms relative to the fixed ring frame, not the Euler angle rotation about Z . For example, Figure 3 (1) is the arm with a 90° orientation to FR.

Now let pFR=[x,y,z]T denote the position vector of the center of the moving ring platform expressed in the fixed ring frame. This is obtained directly from the commanded pose input to the inverse kinematics.

${\text{Using}}^{FR}p$ and the rotation matrix in Equation 1, we can compute the moving-platform attachment offsets expressed in the fixed frame. First rotate the MR offsets into FR using Equation 3.

$${\;\;}^{FR}b_{i}={\;\;}^{FR}\;R_{MR}\;{\;\;}^{MR}b_{i}$$ (3)

The global position of the i -th moving attachment point is computed using Equation 4.

$${\;\;}^{FR}r_{i}={\;\;}^{FR}p+{\;\;}^{FR}b_{i}.$$ (4)

Now that we have all vectors in the **FR** coordinate space, each arm vector ${\;\;}^{FR}d_{i}$ (from base point $A_{i}$ to moving point $B_{i}$ ) can be calculated using Equation 5.

diFR=riFR−aiFR=pFR+RMRFR biMR−aiFR. (5)

Or equivalently, in component form,

diFR=x+bix−aixy+biy−aiyz+biz−aiz=x−xiy−yiz−zi,di=‖diFR‖. (6)

The Euclidean distance formula in Equation 7 gives the required linear actuator length.

$$d_{i}=\sqrt{{\left(x-x_{i}\right)}^{2}+{\left(y-y_{i}\right)}^{2}+{\left(z-z_{i}\right)}^{2}}.$$ (7)

$$\left\{\begin{array}{cc} x_{i} & =g\cos\gamma_{i}-h\left(\cos\gamma_{i}\;r_{11}+\sin\gamma_{i}\;r_{21}\right),\\ y_{i} & =g\sin\gamma_{i}-h\left(\cos\gamma_{i}\;r_{12}+\sin\gamma_{i}\;r_{22}\right),\\ z_{i} & =-h\left(\cos\gamma_{i}\;r_{13}+\sin\gamma_{i}\;r_{23}\right), \end{array}\right.$$ (8)

A compact expression for the intermediate terms is given in Equation 8, where rjk are the elements of the rotation matrix FRRMR in Equation 1. These intermediate terms can be seen in Figure 4B.

$d_{i}$ tells us the length of each arm, and utilizing the linear pitch we can then compute the angles needed for the small motors. As the pitch tells us for every 1 mm to move, how many rotations to perform. However, the large motor angles and the passive joint angles still need to be computed. We note the physical constraints placed on these parameters shown in Table 2.

**TABLE 2:** Kinematics output constraints for Robossis V2. Limits are imposed by mechanical design and hardware travel, not by the inverse-kinematics formulation.

Constraint $C_{i}$	Limit (Robossis V2)
Large motor angles $\theta_{i}$	$|\theta_{i}|\leq 30{}^{\circ}$
Spherical joint angles $A_{i}$	$|A_{i}|\leq 30{}^{\circ}$
Linear actuator stroke $s_{i}$	$0\leq s_{i}\leq 200$ mm
Passive joint angles $\psi_{i}$	$|\psi_{i}|\leq 30{}^{\circ}$

We can represent the arm direction in a local coordinate frame $C_{i}$ , whose origin is located at $A_{i}$ . In this frame, we parameterize the arm direction using the active rotary motor angle $\theta_{i}$ (large motor) and the passive joint angle $\psi_{i}$ :

diCi=disinψi−sinθi cosψicosθi cosψi. (9)

We can then transform this vector into the fixed ring frame using

diFR=RCiFR diCi. (10)

Where RCiFR is a rotation about the fixed z -axis by $\gamma_{i}$ :

RCiFR=cosγi−sinγi0sinγicosγi0001. (11)

By replacing Equations 9, 11 into Equation 10, and using Equation 6, $\psi_{i}$ and $\theta_{i}$ can be calculated using Equations 12, 13, respectively.

$$\psi_{i}=\sin^{-1}\;\left(\frac{\cos\gamma_{i}\;\left(x-x_{i}\right)+\sin\gamma_{i}\;\left(y-y_{i}\right)}{d_{i}}\right).$$ (12)

$$\theta_{i}=\sin^{-1}\;\left(\frac{\sin\gamma_{i}\;\left(x-x_{i}\right)-\cos\gamma_{i}\;\left(y-y_{i}\right)}{d_{i}\cos\psi_{i}}\right).$$ (13)

Thus, we have computed the large motor angles and the passive joint angles. These angles rotate around $n_{i}$ , which is orthogonal to the motor shaft axis, as illustrated in Figure 4. To compute spherical-style direction angles for the arm in the fixed ring frame, first form the unit direction vector by applying Equation 14.

 FRd^i=diFRdi=d^ixd^iyd^iz. (14)

The components ${\hat{d}}_{ix},{\hat{d}}_{iy},{\hat{d}}_{iz}$ are the dot products of ${\;\;}^{FR}{\hat{d}}_{i}$ with the fixed ring unit axes (i.e., direction cosines). In Robossis V2, we define a spherical joint angle $A_{i}$ as the angle between the arm unit direction and a reference unit normal $\hat{n}$ , computed using Equation 15.

$$A_{i}=\cos^{-1}\;\left(\hat{n}\cdot {\;\;}^{FR}{\hat{d}}_{i}\right).$$ (15)

Here, $\hat{n}$ is a unit normal vector (i.e., $\|\hat{n}\|=1$ ) that represents the reference direction used to measure arm tilt. In our implementation, $\hat{n}$ is obtained by normalizing the cross product of two non-collinear reference vectors, as shown in Equation 16. These vectors are $b_{1}$ and $b_{2}$ , which are the vectors from the center of the moving ring to two of the moving attachment ring point $B_{i}$ .

$$n=b_{1}\times b_{2},\;\hat{n}=\frac{n}{\left\|n\right\|}.$$ (16)

### 2.6 Testing methodology

#### 2.6.1 Workspace analysis methodology

Two common workspace definitions are considered: the *translational workspace*, where the end-effector orientation (α,β,γ) is held fixed, and the *rotational workspace*, where each position must remain reachable across a range of orientations. In general, the rotational workspace is a subset of the constant-orientation workspace, since allowing rotation causes the robot to encounter joint and actuator constraints sooner, reducing the set of reachable positions. Baseline translation and rotation ranges of Robossis V2 are depicted in Table 3.

**TABLE 3:** Limits of Robossis V2’s constrained workspace. Rotation ranges listed are considered at the midrange positions, (0 mm, 0 mm, 470 mm) and (0 mm, 0 mm, 284 mm) for Robossis V2 and Robossis V1, respectively.

Motion	Robossis V2 range	Robossis V1 range
x (mm)	−200→200	−144→146
y (mm)	−200→200	−144→146
z (mm)	365→575	214→354
α (deg)	−27→27	−21→23
β (deg)	−27→27	−22→22
γ (deg)	−90→90	−44→44

For the translational workspace, the clinically required translational range is mapped into the numerical analysis as a cylindrical volume derived from clinically-determined requirements shown in Table 4. The translational workspaces are sampled using a spatial step size of 2.5 mm in the axial and radial directions and an angular step of 0.05 π rad ( ≈ 9 ° ) in the polar sweep. For the Robossis V2 workspace, candidate Cartesian points are sampled on a 5 mm grid with x,y∈[−575,575] mm and z∈[182.5,1150] mm, while the orientation is fixed at α=β=γ=0° . A point is accepted only if all actuator lengths satisfy $|d_{i}-470|\leq 105$ mm and all spherical joint angles satisfy $|A_{i}|\leq 30{}^{\circ}$ .

**TABLE 4:** Required ranges for femur fracture alignment based on prior clinical studies (Citak et al., 2011; Lowe et al., 2022; Kim et al., 2017; Bråten et al., 1993). The values reflect measured translational and rotational deviations between the proximal and distal fracture fragments, characterizing the extent of displacement commonly observed in femoral fracturepresentations.

Motion type	Anterior–Posterior axis	Medial–Lateral axis	Femoral–Shaft axis
Translations	±1.89 cm	±4.83 cm	±5.4 cm
Rotations	±10.3°	±8.1°	±42.0°

Algorithm 1 **Pseudocode for numerical evaluation of the translational workspace using inverse kinematics and constraint validation.**

1: **for** $x=x_{\min}$ to $x_{\max}$ **do**
2:  **for** $y=y_{\min}$ to $y_{\max}$ **do**
3:   **for** $z=z_{\min}$ to $z_{\max}$ **do**
4:    Define candidate pose (x,y,z,α,β,γ)

5:    Compute inverse kinematics
6:    **if** all actuator and joint constraints are satisfied **then**
7:     Store (x,y,z) as a valid workspace point
8:    **end if**
9:   **end for**
10:  **end for**
11: **end for**

For the rotational workspace, the robot is evaluated at a fixed midrange Cartesian position, and the orientation variables are sampled directly in orientation space. The rotational coordinates are discretized at 1° increments over the prescribed angular ranges, and each sampled orientation is evaluated using inverse kinematics subject to the same actuator stroke and joint-angle constraints. Valid orientations are mapped into a 3D point cloud in rotational space, defining the rotational workspace envelope. The clinically required rotational range is represented in Table 4, and is sampled on the same 1° grid. In both cases, workspace boundaries are reconstructed using an alpha-shape method, providing a consistent approximation of the workspace envelope for comparison with the clinically required range.

#### 2.6.2 NDI Polaris Vega XT camera

The external optical metrology system that we utilize is the NDI Polaris Vega XT. This camera utilizes triangulation to compute the origin of a rigid body in the perspective of the camera. We use these reported coordinates to compute a position in our desired frame mentioned above. The tracking volume of the camera begins in the space 950 mm in front of the camera, and the initial pyramid volume exists in the space between the distances of 950 mm and 2400 mm. The camera also has an extended pyramid volume which exists in the space between the distances of 2400 mm and 3000 mm. The NDI Polaris Vega XT is factory-calibrated, meaning its intrinsic parameters and sensor geometry are predefined by the manufacturer, eliminating the need for user-performed calibration that could introduce additional sources of error. This ensures a consistent and reliable measurement setup across experiments. Consequently, the measurement uncertainty associated with the optical tracking system is governed by the manufacturer-specified accuracy of 0.12 mm within the initial tracking volume and 0.15 mm within the extended volume (Northern Digital Inc, 2026). The sampling interval is 100 ms, and the positions were logged after reaching a given point.

### 2.6.3 Trajectory testing methodology

To facilitate independent kinematic validation of the Robossis end-effector, an external optical metrology system was employed. A set of retro-reflective markers was mounted on the circumference of the Moving Ring (MR), forming a rigid body that was tracked in real time using an NDI Polaris optical sensor. As shown in Figure 3 (2), three of these markers are arranged to form an equilateral triangle, enabling estimation of the MR center. Two additional asymmetric markers complete the five-marker rigid body, providing the NDI camera with the asymmetry required for unambiguous pose estimation.

To express measurements from the Polaris coordinate frame in the robot fixed ring (FR) frame, a one-time registration procedure was performed at the home configuration. The robot was moved to its predefined home pose, corresponding to a known MR pose of (0,0,465,0,0,0) in the FR frame. At this configuration, both the transformation from FR to ${\mathrm{MR,}\;}^{FR}T_{MR}$ , and the transformation from the Polaris frame to ${\mathrm{MR,}\;}^{\mathrm{NDI}}T_{MR}$ , are known. These relationships allow computation of the constant ${\text{transformation}}^{FR}T_{\mathrm{NDI}}$ , which maps measurements from the Polaris frame to the robot frame. If the camera position changes, this transformation must be recomputed; however, during testing, the camera remained fixed. This method also allows the use of a single rigid body and enables efficient repetition of experiments.

Once this transformation is established, all subsequent measurements of the MR pose can be expressed in the FR frame by applying Equation 17.

TMRFR=TNDIFR⋅TMRNDI (17)

Note that the MR rigid body is defined such that its origin coincides with the geometric center of the moving ring. Therefore, no additional offset transformations are required during the experiments. As a result, the overall registration error is primarily governed by the optical tracking system. Specifically, it arises from two main sources: the initial estimation of the TNDIFR transformation at the home configuration, and the measurement error associated with tracking the MR rigid body.

In a surgical setting, the required accuracy is derived from the Thoresen metric, which evaluates post-operative alignment. To be classified as “Excellent”, the final result must exhibit less than 1 cm of translational displacement and less than 5 ° of angular deformity (Yu et al., 2007). The objective of Robossis is to achieve and surpass this threshold, thereby avoiding misalignment, which is typically defined as angular deviations exceeding 5 ° (Honeycutt et al., 2019).

Table 4 highlights the typical range needed for alignment of femur fractures. The design objective of Robossis is to operate across this full translational and rotational range while achieving sub-millimeter and sub-degree precision.

## 3 Results

### 3.1 Workspace

#### 3.1.1 Translational workspace

**FIGURE 5:**
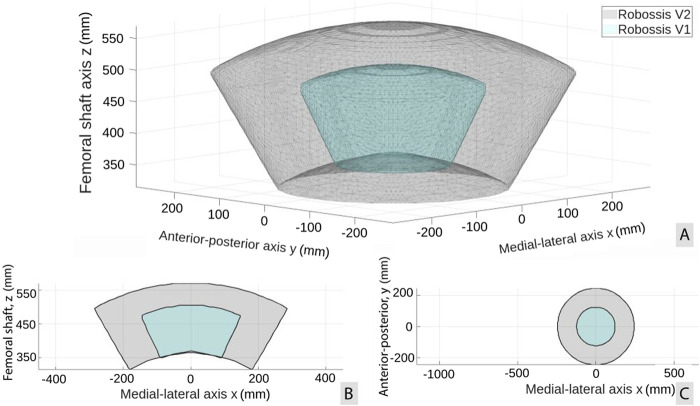
**(A)** The constrained translational workspaces for Robossis V2 and its predecessor V1 and overlaid for comparison. No rotational movement is included for either version. **(B)** A 2D slice of the translational workspaces at y = 0 mm. **(C)** A 2D slice of the workspaces at z = 455 mm.

Figure 5 characterizes the translational capabilities of Robossis V2. Notably, the rate of volumetric expansion varies based on the direction of approach, with a more gradual gradient observed from the distal end compared to the rapid convergence from the proximal boundary. The Home Configuration is calibrated to (0 mm, 0 mm, 465 mm, 0 ° , 0 ° , 0 ° ) to balance reachability with clinical safety. Although Z=490 represents the absolute peak of the translational cross-section, the selected home position provides the necessary margin to mitigate the vast majority of malalignment. This setup also offers better equilibrium, as Robossis can now move 100 mm in both directions from its Z=465 center point.

The average human femur size is approximately 436.88 mm for males and 402.38 mm for females (Moosa et al., 2021). In a supine patient configuration with no leg tilt, Robossis V2 can reach approximately 50% of the femur bone itself. Because the bone is drilled and rigidly attached at the base during the setup workflow, this range is sufficient for Robossis to perform alignment without workspace constraints. The critical factor for Robossis is the relative displacement between the two fragments rather than the total bone length. Consequently, even if fragments were displaced by half the total femur size, Robossis would maintain sufficient reach to complete the reduction.

As shown in Figure 5, the V1 model exhibits a translational workspace approximately six times smaller than its successor. This limitation stems primarily from the proximal positioning of the moving ring relative to the actuation pivots. The workspace is constrained because the linear actuators reach their maximum extension at a displacement equal to the arm length. This relationship can be visualized by analogy to a flashlight: for a fixed pivot, the linear displacement at a distant target is larger than that at a nearby one. In this system, the motor joints serve as the pivots. The V2 design positions the moving ring farther from these pivot points, thereby amplifying its effective range of motion for a given actuator stroke. Consequently, although both models share the same nominal radial dimension, the V2 configuration achieves significantly greater displacement in the X and Y directions for equivalent offsets along the Z-axis.

#### 3.1.2 Rotational workspace

**FIGURE 6:**
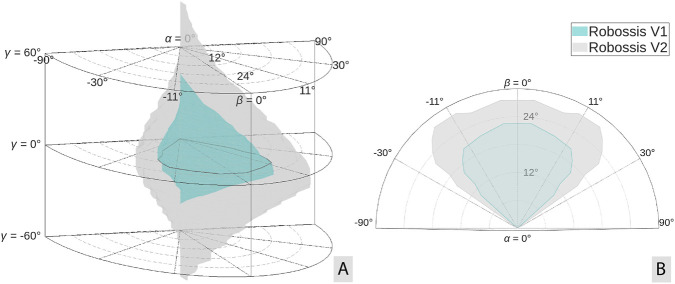
**(A)** The rotational workspaces for Robossis V1 and V2 are overlaid for comparison. Both mechanisms are at their midrange position, (0 mm, 0 mm, 284 mm) and (0 mm, 0 mm, 470 mm), for Robossis V1 and Robossis V2, respectively. **(B)** A 2D slice of the rotational workspaces at γ = 0°.

As illustrated in Figure 6, the Robossis V2 mechanism exhibits a substantially expanded rotational workspace in comparison to its predecessor. This improvement indicates a considerable increase in orientation flexibility, which is critical for surgical manipulation in confined anatomical environments. Consistent with the translational workspace analysis, the configuration yielding the highest degree of rotational dexterity is centered near the optimal operating pose for rotations, which is located at (0 mm, 0 mm, 470 mm, 0 ° , 0 ° , 0 ° ). Within the Robossis coordinate convention, the rotational parameters correspond to α as pitch, β as yaw, and γ as roll. Thus, the enlarged workspace observed in V2 reflects a significantly improved capability to pitch, yaw, and roll the surgical end-effector, ultimately enhancing the robot’s dexterity and operational versatility.

### 3.2 Robossis V2 and the Gough-Stewart Platform

The performance of Robossis was benchmarked against the 3_3-SP S-GSP architecture (Dasgupta and Mruthyunjaya, 2000). This analysis was performed for both translational and rotational workspaces. We observed that the translational workspace of Robossis V2 is more than ninety times larger than that of the GSP. The constrained translation and rotational workspaces for Robossis V2 and the GSP were analyzed alongside the literature data presented in Table 4 to provide a clinically focused perspective on the capabilities of each system.

#### 3.2.1 Required translational range for alignment

**FIGURE 7:**
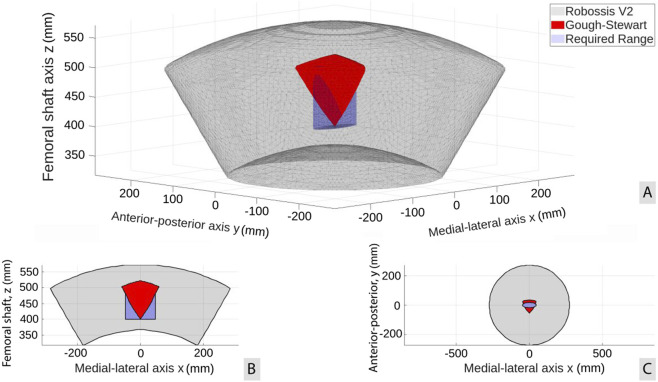
**(A)** The constrained translational workspaces of Robossis V2 and the Gough-Stewart Platform are overlaid for comparison, accompanied by the translational clinically required range for femur fracture alignment. **(B)** A 2D slice of the translational workspaces at y = 0 mm. **(C)** A 2D slice of the translational workspaces at z = 455 mm.

Figure 7 presents the constrained translational workspaces for Robossis V2 and the GSP, accompanied by the required translational range for femur fracture alignment detailed in Table 4. Robossis V2 can fulfill the required range with substantial coverage outside, while the GSP does not reach the clinical requirements for this range.

#### 3.2.2 Required rotational range for alignment

**FIGURE 8:**
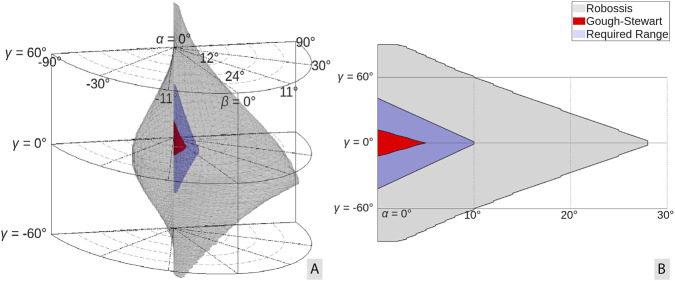
**(A)** The constrained rotational workspaces of Robossis V2 and the Gough-Stewart Platform are overlaid for comparison, accompanied by the rotational clinically required range for femur fracture alignment. Both mechanisms are at their midpoint, (0 mm, 0 mm, 470 mm) and (0 mm, 0 mm, 362 mm), for Robossis V2 and the GSP, respectively. **(B)** A 2D slice of the rotational workspaces at β = 0°.

Figure 8 presents the constrained rotational workspaces for Robossis V2 and the GSP, alongside the required rotational range for femur fracture alignment specified in Table 4. Here we see that Robossis V2 can fulfill the clinical requirements for correction on all axes, while the GSP is severely limited across all rotational movements.

### 3.3 Trajectory testing

#### 3.3.1 Degree of freedom (DOF) testing

**FIGURE 9:**
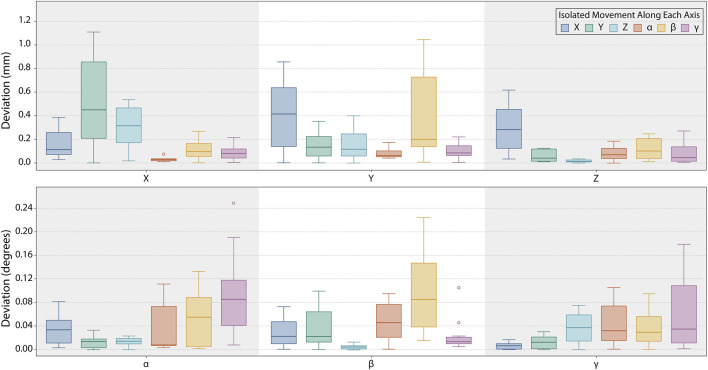
Absolute translational and rotational deviations for each axis. The first row shows the measured deviations in X, Y, and Z when each degree of freedom was commanded independently. The second row presents the corresponding deviations in each rotational degree of freedom when each degree of freedom was utilized. For example, the X-subplot depicts the deviations when moving in X, Y, Z, α , β , and γ one at a time.

This test involved actuating each degree of freedom of Robossis independently. For example, the system was commanded to translate along the X-axis, return to the home configuration, then translate along the Y-axis, and so forth. For each degree of freedom, Robossis was commanded to incremental positions, and upon completion of each increment data was retrieved. The purpose of this test was to determine whether the robot’s physical axes were properly aligned with its defined kinematic coordinate frame. Figure 9 illustrates the resulting spread of absolute deviations for each isolated movement.

#### 3.3.2 Spiral trajectory

**FIGURE 10:**
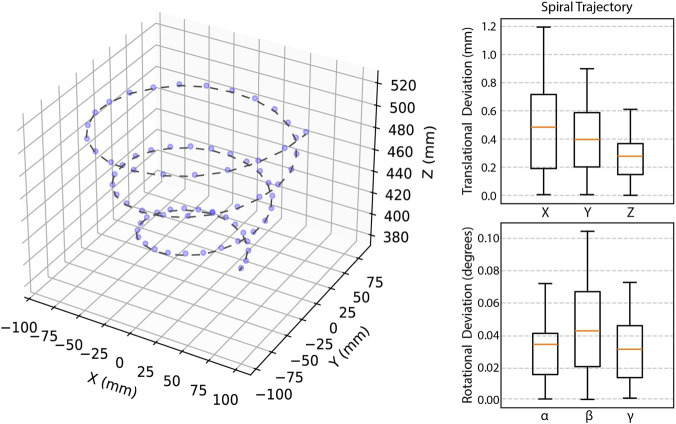
Spiral motion test results. The blue dashed line is the commanded motion, while each dot represents a logged position. The right hand side depicts the deviations in the translations and rotations observed during the trajectory.

After performing rotational and translational testing separately, Robossis was commanded to follow a combined translational trajectory forming a spiral, as shown in Figure 10. This path was selected because it continuously varies both direction and curvature, creating a more demanding reference than piecewise-linear motions. Additionally, the spiral provides a uniform sweep of the workspace, enabling performance comparisons across regions where actuator leverage and kinematic coupling may differ. The primary objective of this test was to quantify tracking accuracy when multiple translational axes are commanded simultaneously, thereby evaluating coordinated multi-axis motion rather than isolated single-axis performance. This trajectory also serves as a sensitivity check for error accumulation during continuous motion, helping determine whether deviations grow as internal actuators execute coupled movements over time.

#### 3.3.3 Repetition testing

**FIGURE 11:**
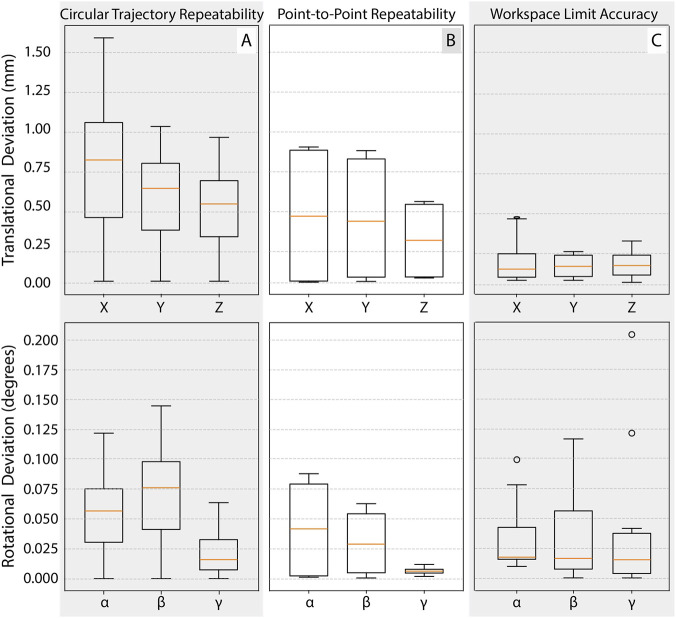
End-effector positioning accuracy under repeatability and required workspace range. Translational (top row) and rotational (bottom row) deviations measured during **(A)** point-to-point repeatability between a predefined target pose (80 mm, 100 mm, 510 mm; 10°, 10°, 10°) and the home position (0 mm, 0 mm, 465 mm, 0°, 0°, 0°), **(B)** circular trajectory repeatability, and **(C)** the required alignment workspace testing within clinically required femoral alignment ranges.

An additional evaluation was repetition testing, in which two trajectories were assessed and the results were summarized as box-plot panels in Figures 11A,B. The first test entailed moving to the position (80 mm,100 mm,510 mm,10°,10°,10°) and then returning to the home position repeatedly (Figure 11A. The goal of this test was to quantify point-to-point accuracy while utilizing all 6 degrees of freedom. A second repetition test was performed using a circular trajectory, where Robossis was commanded to execute the circle 20 times while logging the resulting points (Figure 11B). This was designed to identify any variation between repeated executions of the same commanded path.

#### 3.3.4 Required alignment workspace testing

The required alignment testing consisted of moving the robot to the extremes of the required ranges for femur fracture alignment (Table 4), with performance summarized as a box-plot panel in Figure 11C. This test was of particular importance in determining whether Robossis can achieve sub-millimeter and sub-degree performance within a clinically relevant range.

## 4 Discussion

### 4.1 Analysis of results

Overall, the results indicate that Robossis V2 is both highly precise and capable of operating well beyond the clinically required alignment range. The workspace volume comparisons (Table 5) show that the constrained translational workspace of Robossis V2 is approximately 136 times larger than the translational range required for femur fracture alignment, and the constrained rotational workspace is approximately 79 times larger than the required rotational range. Relative to prior mechanisms, Robossis V2 provides a substantial increase in reachable volume: its translational workspace is approximately 5.9 times larger than Robossis V1 and approximately 93 times larger than the GSP benchmark, while its rotational workspace is approximately 4.3 times larger than Robossis V1 and over 1100 times larger than the GSP (Table 5). Collectively, these results confirm that Robossis V2 covers the clinically relevant alignment range with significant margin, providing flexibility for patient setup and intraoperative variability.

**TABLE 5:** Translational and Rotational workspace volumes of the three analyzed mechanisms alongside the corresponding volumes of the required range of movement for femur fracture alignment detailed in Table 4.

Volume type	Robossis V2	Robossis V1	GSP	Required range
Translational ( ${\text{cm}}^{3}$ )	39223.67	6628.04	421.06	287.55
Rotational ( ${\text{deg}}^{3}$ )	14176.90	3309.42	12.39	178.51

Trajectory testing confirmed that the clinically required workspace is fully usable, with consistently high positional precision throughout. All deviations are reported as absolute errors—representing the total magnitude of difference between commanded and measured pose—and therefore reflect overall positioning error rather than directional bias. Across all trajectory data collected, the 75th percentile of both translational and rotational errors remained sub-millimeter and sub-degree, respectively, demonstrating that Robossis V2 achieves the target precision across the vast majority of operating conditions. The required workspace evaluation is of particular clinical significance, as it assessed system performance within representative alignment ranges rather than theoretical extremes. Robossis V2 maintained sub-millimeter and sub-degree behavior across most measurements within this envelope, reinforcing its suitability for real-world use. While minor axis coupling was observed during the degrees-of-freedom tests, its magnitude was small relative to clinical tolerances and had no meaningful impact on repeatability; repeated trajectories exhibited proportional and consistent behavior across all trials. This coupling was attributed to the optical tracking geometry: the NDI camera’s preference for asymmetric marker configurations meant that the three equilateral markers forming the base of the rigid body introduced a condition in which the camera’s reference axes could not be made fully coincident with the MR frame axes, despite the transformation we introduced. The two asymmetric markers completing the five-marker rigid body were insufficient to resolve this coupling, as the equilateral geometry of the primary three markers continued affect the tracking behavior. Overall, these results establish Robossis V2 as a clinically promising platform for femur fracture reduction—one that combines a large, representative workspace with reliable sub-millimeter and sub-degree precision across the full range of evaluated conditions.

In addition to the 75th percentile, the median absolute translational and rotational errors across the DOF trajectory data ranged from 0.273 mm to 0.484 mm and 0.03 ° to 0.04 ° , respectively. The corresponding 95th percentile errors ranged from 0.522 mm to 0.969 mm for translation and 0.064 ° to 0.092 ° for rotation. Similar results were observed across the other tests.

Although the reported experiments were conducted under unloaded conditions, it is important to consider performance under realistic surgical loading. During femoral fracture reduction, Robossis is expected to experience significant traction forces and torques due to surrounding musculature and soft tissue interaction. These external loads may introduce additional error relative to the unloaded case; however, Robossis was explicitly designed to operate under such conditions.

In particular, the selection of a parallel kinematic architecture was motivated by its inherently higher structural stiffness and load-bearing capability compared to serial manipulators. This design choice allows forces to be distributed across multiple limbs, reducing localized deformation and improving resistance to load-induced deflection. The actuators in Robossis are equipped with encoders, ensuring that commanded joint positions are accurately achieved even under load. Therefore, any residual error at the end-effector is expected to arise primarily from structural compliance or deformation rather than control inaccuracies. While such effects are expected to be limited due to the system’s mechanical design and material properties, future work will focus on quantifying load-dependent error and incorporating model-based compensation strategies (Singh et al., 2024).

### 4.2 Conclusion

This work presented an experimental validation of Robossis V2, a 3-armed, 6-DOF parallel robotic system designed to assist femoral fracture reduction by enabling controlled, high-precision manipulation of the distal fragment. A numerical workspace analysis demonstrated that Robossis V2 substantially exceeds the clinically required alignment range: its constrained translational and rotational workspaces are approximately 136 times and 79 times larger than the required ranges, respectively, and it provides major improvements over both Robossis V1 and the GSP benchmark (Table 5). Independent optical tracking experiments further verified that this workspace is usable with high precision during commanded trajectories. Using absolute deviations between measured and commanded MR pose, the 75th percentile of errors across all collected data remained sub-millimeter in translation and sub-degree in rotation within the clinically relevant workspace. These results satisfy (and surpass) the Thoresen criteria for an “Excellent” outcome and support Robossis V2 as a clinically promising platform for reproducible, high-accuracy femur fracture alignment. Future work will focus on continued evaluation of Robossis V2 in close collaboration with orthopedic surgeons to further refine its role as an assistive system. In particular, we aim to advance surgeon–robot collaborative operation through improved intuitive control and workflow integration. Ongoing cadaver and simulation-based studies will guide iterative design improvements to better address the practical needs of surgeons across diverse fracture scenarios.

## Data Availability

The raw data supporting the conclusions of this article will be made available by the authors, without undue reservation.
